# Trajectories of Psychotropic Medications Before and After an Autism Diagnosis Vary by Age and Sex

**DOI:** 10.1192/bjo.2024.698

**Published:** 2024-08-01

**Authors:** Sébastien Brodeur, Isabelle Dufour, Yohann Chiu, Josiane Courteau, Mélanie Couture

**Affiliations:** 1 Laval University, Faculty of Medicine, Quebec City, Canada; 2 Institute of Psychiatry, Psychology & Neuroscience, King's College London, London, Canada; 3 Nursing School, Sherbrooke University, Sherbrooke, Canada; 4 Sherbrooke University, Sherbrooke, Canada; 5 CHUS Research Center, Sherbrooke, Canada

## Abstract

**Aims:**

Interventions to support people with autism are multidimensional, but primarily psychosocial in nature. These interventions include behavioural, educational and support therapies. Some psychotropic medications are used to manage medical and psychiatric comorbidities associated with autism, which interfere with daily social and occupational functioning or limit the implementation of psychosocial interventions. The aim of this study is to describe the trajectories of psychotropic medications in people newly diagnosed with autism according to sex and age.

**Methods:**

This is a retrospective cohort study based on medico-administrative data from the Régie de l'assurance santé du Québec. The cohort included all people living in the province of Quebec (Canada) with a first diagnosis of autism (incident cases) recorded during hospitalisation or during a medical visit between January 2012 and December 2016 (index date: first diagnosis). Only individuals covered by the public prescription drug insurance plan one year before and one year after the index date were included. A patient was considered exposed to a drug from the date a prescription was claimed at a community pharmacy and for the time the drug was provided. However, as no information was available on inpatient drug, the drug trajectory represents the outpatient drug trajectory. The five classes of psychotropic drugs considered were: 1) anticonvulsants and mood stabilisers; 2) antipsychotics; 3) antidepressants; 4) anxiolytics/hypnotics; and 5) psychostimulants. Drug trajectories are represented using state sequence analyses.

**Results:**

The study cohort included 3284 people, of which 867 (26.4%) were females and 2417 (73.6%) were males. Overall, 51.6% of the cohort claimed a psychotropic medication in the year preceding diagnosis and 61.1% in the following year, with higher proportions among females and increasing with age. Psychostimulants were the most prescribed medications among people diagnosed at ages ≤12 years, while antipsychotic use increased considerably with age, becoming the most commonly prescribed medication among those diagnosed in adulthood (≥18 years), with use rates reaching as much as 80% among those diagnosed between 36 and 60 years. State sequence analyses demonstrate slight variations in the use of psychotropic medications over time, but significant variations by age category and sex.

**Conclusion:**

Although psychosocial interventions are recognised by clinical practice guidelines as the cornerstone of interventions for people with autism, the use of psychotropic medications is widespread. This highlights a significant gap between the recommendations of these guidelines and what is observed in the real world.

